# Supplementary figures and images for: Chondrogenic Differentiation of Adipose-Derived Stromal Cells Induced by Decellularized Cartilage Matrix/Silk Fibroin Secondary Crosslinking Hydrogel Scaffolds with a Three-Dimensional Microstructure
Source: Polymers (Basel). 2023 Apr 13;15(8):1868. doi: 10.3390/polym15081868 (PMC10144539; doi:10.3390/polym15081868)

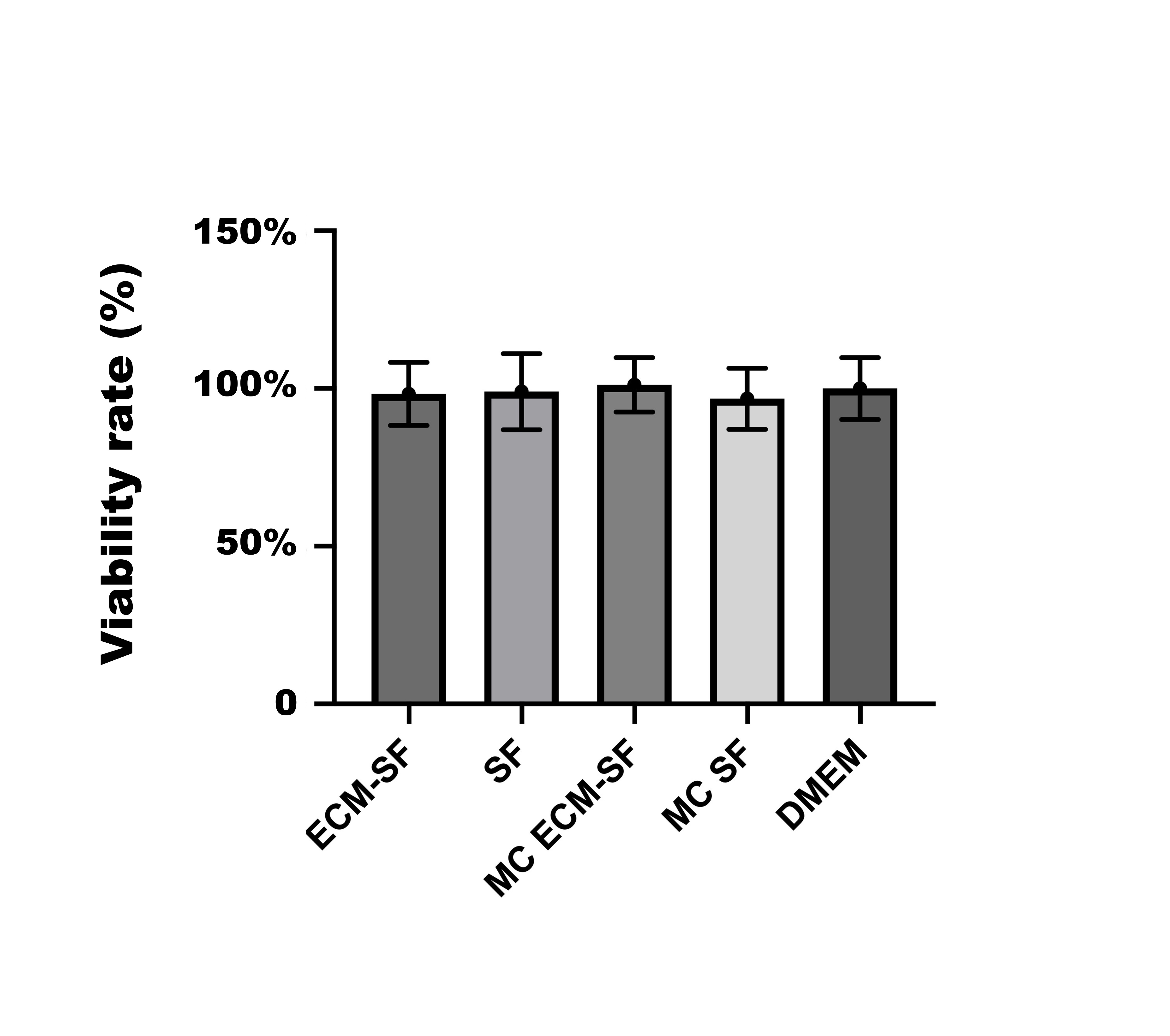

Supplement: Supplementary file 1 [file polymers-15-01868-s001.zip › polymers-2251651-supplementary.jpg]
